# The Osteoimmunologic Basis of Biologic and Bioengineered Scaffolds in Fracture Healing

**DOI:** 10.3390/bioengineering13020223

**Published:** 2026-02-14

**Authors:** Hannah Shelby, Sarah Bergren, Julian Wier, Edward M. Schwarz, Jay R. Lieberman

**Affiliations:** 1 Department of Orthopaedic Surgery, Keck School of Medicine, University of Southern California, Los Angeles, CA 90033, USA; 2 Department of Orthopedics, Center for Musculoskeletal Research, University of Rochester Medical Center, Rochester, NY 14609, USA; 3 Alfred E. Mann Department of Biomedical Engineering, Viterbi School of Engineering, University of Southern California, Los Angeles, CA 90089, USA

**Keywords:** nonunion, fracture healing, osteoimmunology, immunomodulation, BMP, MSC, gene therapy, scaffold

## Abstract

Fracture nonunion or delayed union remains a significant clinical problem that burdens both the patient and the healthcare system. Defined as failure for bone to unite 9 months post injury or 3 months with no progression toward union, the pathology of nonunion may require multiple surgical interventions with associated morbidity. Increasing evidence has highlighted that nonunion is a multifaceted problem, not only a result of mechanical failure, but also a product of persistent dysregulation of the osteoimmune microenvironment manifested as impaired osteogenesis and bone healing. While current approaches focus on enhanced fixation and various bone grafting strategies, these treatments often fail to coordinate healing with osteoimmune regulation. This review summarizes the emerging biologic and bioengineering approaches that target osteoimmunology to enhance fracture repair. Scaffold systems, including metals, bioceramics, hydrogels, and micro/nanoparticle formulations, are being increasingly engineered to provide structural support while directing macrophage polarization and stimulating osteogenic signaling. We also review cell-based therapies and gene-modified constructs that are being developed to introduce osteoimmunology cues that halt chronic inflammation and promote an osteogenic microenvironment.

## 1. Introduction

Osteoimmunology posits an interplay between the immune system and bone cells that has functional consequences on the skeleton [1]. Research over the past 25 years has substantiated this concept and demonstrated the impact of osteoimmunology on multiple pathologies, including fracture healing, osteoarthritis, ankylosing spondylitis, osteoporosis, and rheumatoid arthritis [2,3]. Once regarded as distinct domains, bone remodeling and immune regulation are now recognized as intertwined processes sharing common progenitors, signaling pathways, and molecular mediators. The discovery that T cells and macrophages influence osteoclast differentiation through the receptor activator of nuclear factor kappa-Β (RANK)–RANK-ligand axis revealed that bone resorption is not driven entirely by mechanical or endocrine factors, but is also regulated by immune cells [4]. The innate immune system regulates the initiation and resolution of inflammation during bone repair, while adaptive immune cells release cytokines to influence the osteogenic and remodeling phase of fracture healing [5]. Conversely, skeletal cells produce cytokines and growth factors that shape immune responses within the bone marrow and at injury sites [6]. This intricate crosstalk maintains homeostasis under physiologic conditions but becomes maladaptive in chronic inflammatory states, leading to conditions such as delayed fracture healing and nonunion. As such, osteoimmunology provides a unifying framework for understanding how inflammation influences the balance between bone formation and resorption, and offers a mechanistic rationale for emerging therapies that target immune pathways to impact bone healing.

Fracture nonunion, defined as the failure of damaged bone to unite within 9 months post injury or 3 months with no progression toward union, represents a significant healthcare-related burden in both young and older adult populations [7]. Nonunion and delayed union encompass a spectrum of fractures, ranging from those that may simply require a longer time to heal to critical-sized segmental defects that are at high risk of developing into a nonunion [8]. Nonunion may also be associated with a prolonged, unresolved inflammatory state [9]. Here, we review recent advancements and potential therapies to treat nonunions.

### Clinical Relevance and Therapeutic Gaps

Fracture nonunions represent a significant healthcare burden. In 2019, it was estimated that there were more than 150 million new fractures worldwide, a one-third increase from the 1990s [10]. Moreover, 5–10% of all fractures do not heal successfully, leading to nonunion or malunion [11,12]. Inadequately healed fractures impose a notable burden on individuals and the healthcare system, as treatment may require multiple surgeries and lead to an increased risk of further complications, such as infection. These patients also face an increased risk of depression and anxiety related to fear of re-injury, loss of function, and inability to return to normal activities [13,14]. Despite treatment, many may eventually require amputation in cases of recalcitrant nonunion or persistent infection [15]. Nonunited fractures also significantly increase total costs compared to uncomplicated fractures, largely driven by indirect costs, with direct costs averaging $30,000 and indirect costs exceeding $80,000 [16,17].

Delayed union and nonunion are increasingly recognized as not simply mechanical failures but also as diseases of a dysregulated osteoimmune microenvironment [18]. Despite this, current treatment regimens, including internal fixation, bone grafting, and growth factor delivery, have not addressed this essential component of fracture healing biology [19]. Even the most established interventions today demonstrate inconsistent success. Surgical fixation, such as with compression plating or intramedullary nailing, provides mechanical support and allows for physiologic healing, but in complex situations with large bone defects, significant bone loss or fractures lacking biological activity, fixation alone does not provide the necessary cells or signals to allow for sufficient bone repair [20]. Autograft bone, the clinical gold standard, which does offer osteogenic, osteoinductive, and osteoconductive properties, is limited by donor-site morbidity, variable graft quality, and inconsistent success in large or complex defects [20]. Meanwhile, allografts avoid donor morbidity but lack viable cells and critical osteoinductive signals necessary for treating nonunions. Recombinant human bone morphogenetic protein-2 (rhBMP-2), although a powerful osteoinductive factor that stimulates the differentiation of stem cells into osteoblasts, has faced limited adoption due to dose-dependent adverse tissue responses [21]. Its short biological half-life necessitates supraphysiologic local concentrations to achieve sustained signaling, resulting in complications and limiting its clinical utility [22]. Current delivery systems, which release BMP-2 in an initial burst rather than in a controlled, physiologic manner, can result in soft-tissue edema, formation of seromas, and heterotopic bone formation [21].

The challenges associated with the successful treatment of fracture nonunion extend beyond the limitations of grafts and growth-factor delivery and are further exacerbated by the requirement for coordinated immune regulation. Preclinical models highlight that once chronic immune dysregulation is established, purely osteoinductive approaches are insufficient to induce healing. To that end, Cheng et al. observed that delayed BMP-2 treatment in a rat critical-sized defect model yields inferior bone formation and biomechanics despite dose escalation, with non-responders characterized by sustained systemic tumor necrosis factor-alpha (TNF-α)/interleukin (IL)-1β elevation and an unresolved inflammatory signature [23]. Clinical data demonstrate that systemic and local immune phenotypes strongly condition healing trajectories, as patients with elevated terminally differentiated CD8^+^ cells in blood and fracture hematoma have higher rates of delayed healing, with these interferon-gamma (IFN-γ)/TNF-α-producing T cells directly suppressing mesenchymal stem cell (MSC) osteogenesis and survival [24]. Similarly, single-cell profiling of human femoral nonunions revealed an enrichment of monocytes and CD14^+^ dendritic cells, accompanied by a depletion of progenitor myeloid cell types, consistent with chronic inflammation and poor regenerative potential [25]. Proteomic analysis in atrophic nonunion identified upregulation of hepcidin and complement components, implicating persistent innate immune activation and metabolic stress in failed bone repair [25,26]. Large meta-analyses of clinical data demonstrate that inflammatory biomarkers predict a higher risk of nonunion, and analysis of early human callus demonstrates divergent inflammatory phenotype trajectories between union and nonunion within days of injury [27,28]. Attempts to systematically modulate immunity in the setting of fracture repair have shown promise; however, complications related to off-target effects remain a concern. Early low-dose recombinant human TNF-α administration has been shown to amplify the initial neutrophil/monocyte response and improve fracture healing in murine models, while FK506 (Tacrolimus) dampens excessive T-cell-driven inflammation in fractures with severe muscle trauma, enhancing bone repair [29,30]. Collectively, these studies highlight the relevance of targeting immune homeostasis to prevent nonunion. Moreover, these data reveal a critical therapeutic gap and provide a strong clinical rationale for bioengineering approaches that integrate local graft strategies and targeted immunomodulation to recalibrate local osteoimmunology. Together, these data suggest that bioengineering strategies must be designed to actively modify the local and systemic immune environment, modulate disease-specific immune cell interactions, and prevent disease progression rather than merely treating symptoms.

## 2. Immunology and Skeletal Tissue Engineering

Immune cells, including B and T lymphocytes, and osteogenic cells, such as osteoblasts and osteoclasts, share a microenvironment and function together as part of the osteoimmune system [2]. The concept of osteoimmunology was initially developed to understand the role of T cell-mediated osteoclastogenesis in the development of autoimmune arthritis [31]. The immune and skeletal systems share numerous cytokines, growth factors, and signaling molecules such as IL-1, IL-7, and RANKL, which coordinate both bone healing and immune responses [4,32]. Similarly, it has been suggested that osteoprogenitor cells help regulate hematopoietic stem cell populations and that osteoblasts may influence the differentiation of B and T lymphocytes [2].

**Figure 1:**
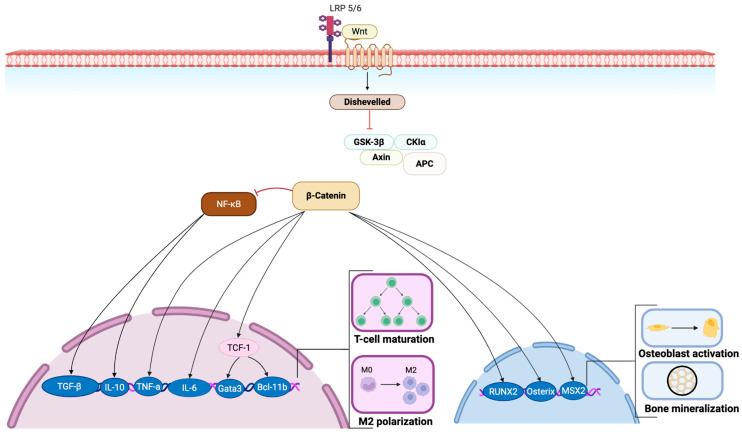
**The Wnt pathway as a shared signaling pathway between the immune system and bone repair.** The Wnt pathway is a key pathway involved in bone repair and immune regulation. Activation of the Wnt pathway leads to inhibition of GSK3β, a β-catenin inhibitor. In immune regulation, β-catenin is involved in promoting the expression of key anti-inflammatory cytokines such as IL-6 and TNF-α, as well as acts as an inhibitor to NF-κB, downregulating the expression of pro-inflammatory molecules such as IL-10 and TGF-β [36]. This ultimately promotes polarization of macrophages towards the M2 phenotype [36]. The Wnt pathway is also involved in T-cell maturation. When β-catenin enters the nucleus of a cell, it binds to TCR-1, which then promotes the expression of genes involved in T-cell development such as BCL11B and GATA3 [37]. For bone repair, BMPs activate the Wnt pathway, which leads to the translocation of β-catenin into the nucleus where it promotes the transcription of osteogenic genes, including Osterix, RUNX2 and MSX2 [34,35]. These molecules influence the development and maturation of osteoblasts and increase bone mineralization.

The immune system and bone repair also share various signaling pathways. The Wnt/β-catenin pathway is involved in the development and regulation of lymphocytes, macrophages and osteogenic cells (Figure 1). In general, activation of the Wnt pathway leads to the activation of the disheveled protein which results in the inhibition of glycogen synthase kinase-3 beta (GSK3β), preventing β-catenin degradation and enabling the translocation of β-catenin to the nucleus where it plays an important role in the transcription of key osteogenic and immune regulatory genes [33,34]. In terms of bone repair, BMPs serve as primary molecular cues that promote this pathway, leading to β-catenin activation and the transcription of genes involved in osteoblast development and bone mineralization such as MSX2, RUNX2 and Osterix [34,35]. The Wnt pathway is also a central regulator of immune cell activation. The Wnt pathway promotes M2 macrophage polarization over M1 polarization through the inhibition of NF-κB, leading to the downregulation of pro-inflammatory genes such as TGF-β and IL-10, and through the stabilization of β-catenin, allowing for the transcription of anti-inflammatory genes including TNF-α and IL-6 [36]. This pathway additionally enhances differentiation of progenitor cells into lymphocytes through the activation of transcription factor TCF-1, which is involved in promoting the transcription of genes necessary for T-cell maturation [36,37]. Likewise, the mTOR pathway is involved in both immune system and bone regeneration signaling as this pathway is a prominent regulator of osteogenic and immune cell growth, proliferation, and differentiation [38].

Importantly, the immune system plays a crucial role in fracture healing, and immune system dysregulation can lead to inadequate bone repair, chronic inflammation, and the development of nonunion. Therefore, understanding the interaction between immune and osteogenic cells has direct clinical applicability, illuminating potential therapeutic strategies targeting immune system modulation to enhance fracture healing and prevent nonunion.

## 3. Inflammatory Phase of Fracture Healing

**Figure 2:**
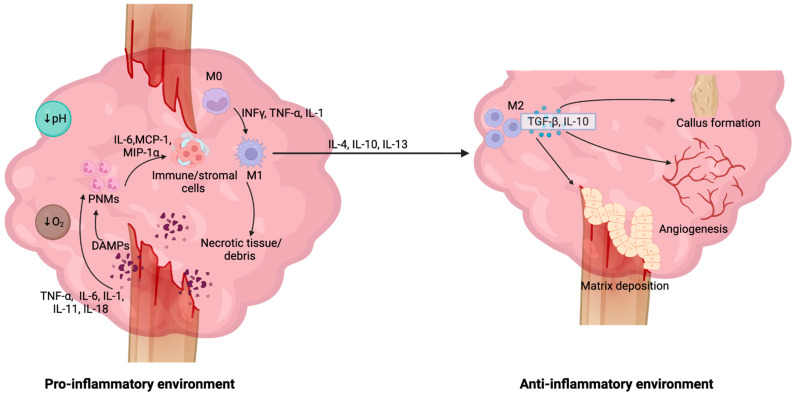
**Immune regulation in fracture healing.** During the acute phase of fracture healing, a reduction in pH and hypoxic conditions occurs, facilitating the release of inflammatory markers and cellular debris [49]. Damage associated molecular patterns (DAMPs) are released from the fracture site as well as other inflammatory cytokines, including TNF-α, IL-1, IL-6, IL-11, and IL-18 [9]. These, along with DAMPs, recruit neutrophils to the injury site. These neutrophils further secrete inflammatory cytokines such as IL-6, MCP-1, and MIP-1α, which recruit additional immune and stromal cells [50]. As healing progresses, neutrophils are replaced by pro-inflammatory M1 macrophages whose primary function is to sterilize the damaged tissue by phagocytosing microorganisms [9,50]. In normal fracture healing, M1 macrophages transition to M2 macrophages that create an anti-inflammatory microenvironment by secreting TGF-β and IL-10. These M2 macrophages also promote angiogenesis, matrix deposition and callus formation, ultimately resulting in bone repair [51,52,53].

Immunology plays a critical role in bone healing, coordinating the transition from inflammation to regeneration (Figure 2). Following injury, the initial hematoma creates an acidic and hypoxic environment that triggers a rapid, local inflammatory cascade characterized by the release of pro-inflammatory cytokines and angiogenic factors [9,39]. Additionally, TNF-α, IL-1, IL-6, IL-11, and IL-18 are key cytokines that regulate the recruitment and proliferation of macrophages and neutrophils [40]. Following the release of these factors, along with damage-associated molecular patterns (DAMPs), polymorphonuclear neutrophils (PMNs) are the first to migrate to the site [41]. While there, neutrophils secrete chemoattractants such as IL-6, monocyte chemoattractant protein-1 (MCP-1), and macrophage inflammatory protein-1α (MIP-1α) to recruit macrophages and reparative stromal cells, capable of more sustained activity within the inflammatory environment [41].

As the inflammatory phase progresses, macrophages replace neutrophils and become the dominant regulatory cell type [9,40]. Initially, monocytes (M0) differentiate into pro-inflammatory M1 macrophages under the influence of DAMPs, and inflammatory cytokines such as IFN-γ, TNF-α, and IL-1 [41]. These early pro-inflammatory M1 macrophages sterilize the injury site and then give way to M2 macrophages that clear dead and dying cells and debris, and secrete transforming growth factor-beta (TGF-β) and platelet-derived growth factor (PDGF) to recruit MSCs and begin the process of soft callus formation via endochondral ossification [42,43]. These cytokines stimulate chondrocyte differentiation within the granulation tissue, leading to soft callus formation, followed by vascular invasion and mineralization into woven bone [41,44]. In addition, tissue-resident osteal macrophages (osteomacs) lining the periosteum and endosteum regulate type I collagen deposition and mineralization, facilitating direct bone formation [9,45].

The polarization of M1 macrophages to M2 macrophages, driven by IL-4, IL-10, and IL-13, also marks the resolution of early inflammation and the initiation of tissue repair via secretion of IL-10 and TGF-β [9,43]. The local increase in M2 pro-healing macrophages supports angiogenesis, matrix deposition, and remodeling [44,45]. Extracellular vesicles (EVs) derived from naïve or M2 macrophages have been found to enhance osteogenesis, whereas M1-derived EVs inhibit bone formation [40,46]. Experimental disruption of macrophage recruitment and cytokine signaling has been shown to impair fracture repair, underscoring the necessity of the initial inflammatory phase followed by M2-driven resolution for establishing the regenerative microenvironment [45,47].

Conversely, a persistent inflammatory signal, driven by factors such as poor vascularity, infection, extensive tissue injury, or comorbidities such as diabetes and smoking, may convert this regenerative process into a chronic, destructive one [48]. The sustained activation of TNF-α and IL-1β signaling promotes osteoclastogenesis and suppresses osteoblast differentiation, leading to bone erosion and impaired bone repair [41]. Further, prolonged presence of M1 macrophages and the presence of activated T-cells, particularly CD8+ and Th1 subsets, have been found to promote catabolism rather than regeneration [9]. Conversely, regulatory T cells, which are important immunomodulators responsible for secreting the anti-inflammatory cytokines IL-10 and TGF-β, have been found to promote osteogenesis and bone repair [9].

## 4. Therapeutic Options

The current gold standard treatment for nonunion and bone defects is autologous bone graft. However, autografts are limited by donor site morbidity, and harvesting may require a secondary surgery site, further increasing the risk of complications and pain [54,55]. Furthermore, autograft efficacy depends on host factors, with older adults and those with a higher comorbidity burden experiencing decreased graft quality, thereby further increasing the risk of failure. Another consideration is the use of allograft bone, which mitigates the risk of donor site morbidity; however, the major limitation of allografts is that they are osteoconductive but not osteoinductive [54,56]. In addition, neither autograft nor allograft provide sufficient structural and biochemical support, which may be needed to heal large segmental defects [54].

**Figure 3:**
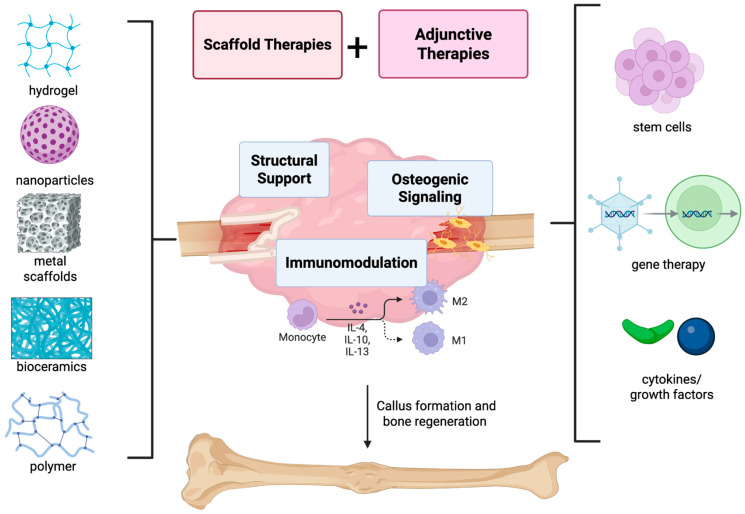
**Osteoimmunomodulatory scaffolds and additional strategies for fracture repair**. Scaffold platforms including meals, bioceramics, hydrogels, nanoparticles, and polymer-based approaches have been able to provide structural support along with immunomodulation, influencing immune cell behavior and promoting osteogenic signaling of the site of fracture [54]. Jointly combining these approaches with adjunctive biologic therapies such as stem cells, gene therapy, cytokines or growth factors have been shown to create a more robust immune and osteogenic response [54]. Taken together, these approaches help transform the local immune response from a pro-inflammatory state toward pro-healing, anti-inflammatory phenotypes, supporting callus formation and bone regeneration.

In the context of bone tissue engineering, a scaffold refers to a three-dimensional (3D) biomaterial structure designed to replicate the native extracellular matrix, providing mechanical support and creating an environment for cellular activities such as cell migration, recruitment, proliferation, and differentiation [57]. Furthermore, scaffolds may guide new tissue formation and act as delivery platforms for bioactive molecules, immunomodulating factors, cells, or gene vectors [57]. Scaffolds can be customized to fit the shape of a defect, as well as exert immunomodulatory effects on the surrounding environment. Biologic scaffolds provide a framework to enhance bone healing and support regeneration. A wide range of scaffold classes exist, including metal-based scaffolds (such as titanium and magnesium alloys), bioceramics which emulate mineralized bone and release bioactive ions, hydrogels that conform to irregularly shaped defects and deliver therapeutics, polymer-based nanoparticles/microspheres allowing for the controlled release of signaling molecules, and hybrid/composite scaffolds that integrate multiple materials to optimize mechanical and biological performance [57] (Figure 3). While a complete engineering description of each scaffold type is beyond the scope of this review, their functional properties and immunomodulating mechanisms are expanded upon in the context of bone healing.

**Figure 4:**
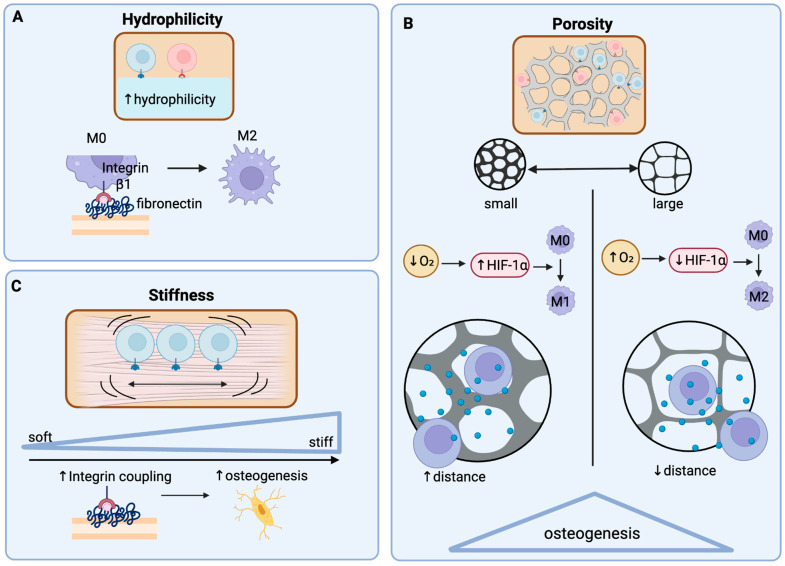
**The properties of scaffold surfaces shape the osteoimmune microenvironment.** The surface properties of scaffolds can influence the behavior and development of cells. In general, the hydrophilicity, porosity, and stiffness of scaffolds play a role in cell adhesion, differentiation and proliferation. (**A**) The hydrophilicity of scaffolds influences the binding of cells. Hydrophobic surfaces can cause the denaturation and conformational change of proteins, which negatively impact cellular binding [65,66]. Hydrophilic scaffolds enable the binding of important proteins with key binding sites for exposed cell integrins, enhancing cell adhesion and ultimately positively impacting cell differentiation and proliferation [65,66]. For example, the adsorption of fibronectin on scaffolds has been found to promote the binding of integrin β1, leading to M2 macrophage polarization and anti-inflammatory cytokine production [65]. (**B**) Porosity also influences the inflammatory environment of cells. With lower porosity, there is less oxygen available for cells which promotes the expression of hypoxia-inducible factor 1-α (HIF-1α), promoting inflammation and the M1 macrophage polarization [67,68]. Conversely, increased porosity results in a decrease in these pro-inflammatory cytokines and enhances the production of anti-inflammatory molecules, shifting the environment to one that is pro-regenerative [54,69]. Further, increased porosity allows for diffusion of paracrine signals from surrounding cells, promoting important immunomodulatory and osteogenic pathways such as the Wnt pathway [70]. (**C**) Scaffold stiffness also plays an important role in cell fate determination and immune regulation. Stiffness is sensed by integrin clustering, which is able to couple extracellular matrix resistance to intracellular cytoskeleton tension [71]. This ultimately activates focal adhesion kinase (FAK), a key regulator in nuclear translocation of mechanosensitive transcription factors [71,72]. These transcription factors modulate osteogenic gene expression and mineralization, as well as M1 to M2 polarization [54,71].

Once implanted, scaffolds interface with the immune system and can be engineered to facilitate the delivery of therapeutic products. These include signaling molecules such as IL-10 or TGF-β1, as well as stem cells or gene vectors that can further modulate the immune system [58]. In addition to serving as a delivery system, scaffolds can provide both mechanical and biochemical support, thereby influencing the local environment. Scaffold surface chemistry and topography regulate immune cell interactions, cell proliferation, and scaffold-cell adhesion, which shape downstream inflammatory and healing pathways [58,59,60]. For example, scaffold roughness can influence M2 macrophage polarization, encouraging an anti-inflammatory microenvironment [61,62]. Likewise, pore size, inter-scaffold networks, and mechanical stiffness can alter the behavior of immune cells and their interactions with surrounding tissues, thereby impacting bone and cartilage regeneration [58]. The hydrophilicity of scaffolds is also important, as modifications of these surface features have been shown to decrease pro-inflammatory cytokine production and increase anti-inflammatory cytokines, while also enhancing cell adhesion and proliferation, thereby providing an ideal environment for tissue repair [58,59,60,63,64] (Figure 4).

Mechanotransduction involving cell–cell and cell–matrix adhesion pathways further influences how implants interact with surrounding tissue, impacting their cellular activity, differentiation, and immune system activation [73]. Integrins and cadherins connect cells to the extracellular matrix and to surrounding cells through their intracellular cytoskeleton, allowing for the regulation of signaling pathways important in tissue repair, such as the Wnt/β-catenin pathway [74,75]. With movement, cells encounter mechanical signals from the surrounding implant (i.e., stiffness, surface topography), and these cell adhesion molecules are uniquely positioned to sense and transduce these cues to modulate cellular responses that influence osteogenesis and osteointegration of implants [75]. Further, mechanotransduction influences macrophage polarization with mechanical stretch and optimal surface roughness, leading to an increase in the pro-healing M2 macrophage phenotype [76,77]. These adhesion-mediated signaling pathways play a role in immune cell migration and retention at the implant surface [78]. Understanding the interplay between mechanotransduction, adhesion pathways, and cellular responses provides a deeper understanding of how implant design and material properties can influence osteoimmunology, offering insight into how these factors impact the local inflammatory environment and support tissue regeneration. Scaffolds have been used in fracture repair and nonunion to provide mechanical support by providing a rigid structure in unstable regions, while also exerting immunomodulatory effects and delivering osteogenic molecules. Moreover, while scaffolds have been shown to have immunomodulatory properties and some osteoinductive capability, scaffolds alone are usually integrated with other therapies to provide further osteoinductive signals necessary for bone regeneration [73,79,80]. Scaffolds can be integrated with stem cells, growth factors, cytokines, and gene vectors to provide osteogenic cells and signals while also engaging in immune regulation to promote a pro-healing microenvironment.

In addition to metals, bioceramics, and hydrogels, thermoplastic polymer-based scaffolds have grown in popularity as an osteoimmunomodulation platform in bone repair. Particularly, biodegradable, thermoplastic polymers such as polycaprolactone (PCL), polylactic acid (PLA), and poly(lactic-co-glycolic acid) (PLGA) have been shown to influence macrophage activity. Widely used in extrusion-based and fused deposition modeling 3D printing, these polymers enable precise control of scaffold architecture, which promote a shift from the pro-inflammatory M1 phenotype toward a pro-healing M2 phenotype [81]. Beyond manipulating scaffold architecture, the immunomodulatory capacity has been further enhanced by including bioactive modification. For example, incorporating immunoregulatory molecules such as magnesium, black phosphorus, or cytokines has been shown to upregulate anti-inflammatory markers while suppressing pro-inflammatory signaling. Using a rat critical-sized calvarial defect model, Liu et al. showed that 3D-printed PCL scaffolds integrated with electrospun microfibers drove macrophage polarization toward an M2 phenotype via PI3K/AKT signaling, resulting in healed defects with increased vascularization and robust bone regeneration on micro-CT and histologic analysis compared to controls [82]. Furthermore, Long et al. demonstrated the in vivo therapeutic efficacy of osteoimmunomodulatory polymeric scaffolds using a distal femoral defect rat model, where 3D-printed PLGA/black phosphorus scaffolds promoted healing of the defect through macrophage M2 polarization, which suppressed inflammatory signaling, and significantly enhanced bone regeneration as confirmed by micro-CT and histological analyses [83].

Even with their ongoing developments, scaffolds are not without their limitations. Many scaffolds, particularly those made from natural biomaterials, lack the mechanical strength necessary for weight-bearing regions [84]. While synthetic-based scaffolds provide mechanical support, the scaffold material itself or its degradation byproducts may not be fully biocompatible, potentially leading to cytotoxicity or an adverse host reaction [84,85]. In particular, hydrolytic degradation of synthetic polymers generates acidic byproducts, including lactic and glycolic acid, which locally reduce pH and promote aseptic inflammation. This acidic microenvironment can trigger foreign body responses and sustain M1 polarization, impairing osteogenesis and limiting MSC survival within the already immune-dysregulated environment characteristic of fracture nonunion [84]. Furthermore, in the context of sterilization, while common methods including gamma irradiation and steam autoclaving generally preserve surface architecture and micron-scale roughness, these processes can alter surface chemistry through oxidation, disruption of polymer chains, or modification of functional groups [86,87,88]. For polymer-based scaffolds, gamma irradiation can induce chain scission or cross-linking, potentially accelerating degradation kinetics and altering local immune responses, while autoclaving may induce hydrolytic or thermal damage that affects surface hydrophilicity and bioactivity [89,90,91]. These considerations should be taken into account to ensure preserved therapeutic potential of the design. Despite these challenges, ongoing advances in biomaterials and scaffold design aim to mitigate toxicity while optimizing structural and biochemical support to promote skeletal tissue repair and counteract the inflammatory environment (Table 1).

### 4.1. Metal-Based Scaffolds

As bone repair is often limited by the surrounding inflammatory environment and mechanical instability, 3D scaffolds containing metal ions such as titanium and magnesium are being increasingly explored for bone tissue engineering due to their inherent mechanical support and immunomodulatory effects. These metals have demonstrated the ability to modify the osteoimmune environment, primarily through enhancing M1-to-M2 macrophage polarization [116]. Beyond their mechanical support, metal implants can be engineered to promote a pro-regenerative, anti-inflammatory environment. Titanium-based implants possess a rough topography that enhances macrophage adhesion. Additional modifications that enhance surface hydrophilicity can promote the regenerative M2 phenotype through upregulating IL-4 and IL-10 and downregulating inflammatory markers such as IL–6 [66,94]. Further, strontium ions within metal scaffolds can increase the osteoprotegerin (OPG):RANKL ratio, shifting the environment away from osteoclastogenesis towards osteoblast activation and bone formation [117,118]. Collectively, these features of metal-based scaffolds make them intriguing for use in fracture and nonunion scenarios, particularly as a way to mitigate the corresponding highly inflammatory microenvironment that can impair bone regeneration.

In fracture repair, prolonged inflammation with insufficient osteogenesis can lead to complications such as nonunion or mechanical failure due to hardware fatigue. Metal-based scaffolds have the potential to provide mechanical support and promote osteogenesis while attenuating the inflammatory environment. In a murine femoral defect model, insertion of a MgZnYNd alloy rod resulted in degradation byproducts, which upregulated IL-10 expression, in turn promoting M2 macrophage polarization and increasing osteogenesis by periosteum stem cells [93]. Similarly, a metal-phenolic network made from tannic acid and strontium layered onto titanium plates was found to improve bone volume and new bone formation about the bone–implant interface in a murine femoral defect model [96]. This surface modification enhanced stem cell recruitment and differentiation, while promoting M2 macrophage polarization [96]. Metal scaffolds have also shown success in healing bone defects in large animal models. Using a sheep mid-diaphyseal tibial defect model, Pobloth et al. sought to investigate the healing potential of 3D titanium mesh-scaffolds. Titanium mesh-scaffolds with lower stiffness led to improved bone healing with faster radiographic healing and more advanced bone formation on histology compared to stiffer scaffolds [97]. Notably, excessive scaffold stiffness promotes stress shielding, which impairs bone formation by attenuating mechanotransductive cues and reducing M2 macrophage polarization, thereby sustaining a pro-inflammatory environment [97,119,120].

**Figure 5:**
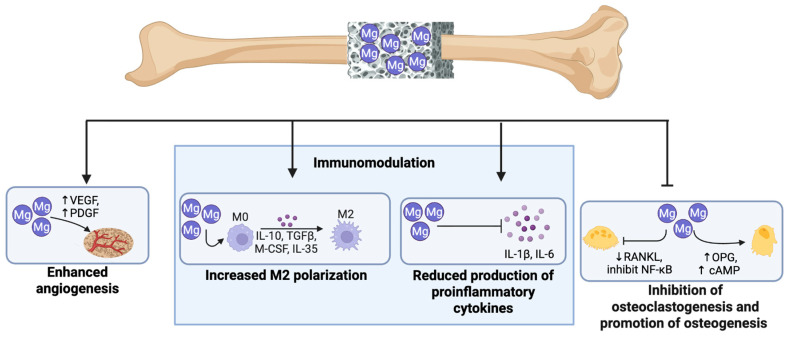
**The osteoimmunological properties of magnesium-based implants.** Magnesium-based scaffolds have a large potential in fracture repair due to magnesium’s ability to enhance osteogenesis and influence the local immune environment. In terms of immunomodulation, magnesium molecules have previously been shown to reduce the production of pro-inflammatory cytokines such as IL-1β and IL-6 [121,122]. Magnesium has also been shown to increase M2 macrophage polarization through mTOR activation, a signaling pathway that plays a role in immune cell activity regulation [123,124]. Further, magnesium promotes bone regeneration and osteointegration of implants through the promotion of osteogenesis and inhibition of osteoclastogenesis. Magnesium is involved in inhibiting the NF-kB signaling pathway and decreases the expression of RANKL, inhibiting osteoclasts and bone breakdown [122,125]. Magnesium has also been shown to increase the expression of cAMP and OPG, which promotes the Wnt/β-catenin pathway and activates osteoblasts, promoting bone formation [121,125]. Additionally, magnesium facilitates angiogenesis, allowing for enhanced blood vessel formation at fracture sites [126,127]. NF-kB, nuclear factor kappa-light-chain-enhancer of activated B cells; OPG, osteoprotegerin; RANKL, receptor activator of nuclear factor kappa-Β ligand.

Magnesium containing scaffolds have shown considerable promise. While other bioinert implants can provide mechanical stability and immunomodulation, magnesium also demonstrates direct osteogenic effects to accelerate union and implant osseointegration [121] (Figure 5). For example, Han et al. demonstrated that a magnesium alloy implant resulted in increased type II collagen deposition at the bone–implant interface compared to titanium implants [98]. Magnesium is thought to act by increasing cAMP within osteocytes, leading to increased activation of the Wnt/β-catenin pathway, resulting in increased osteogenesis and positively influencing implant integration [121]. In a rabbit bone model, magnesium-coated titanium screws were shown to have better osseointegration and increased bone mineralization at both 4 and 6 weeks post implantation compared to titanium alone [95]. Magnesium implants have also been shown to result in reduced stress shielding, as its stiffness is similar to that of cortical bone, allowing for increased movement-stimulated bone healing [121].

The ability of metal-based scaffolds to modulate the inflammatory environment can assist with bone regeneration and the durability of these implants [96]. However, the implantation of these scaffolds presents several challenges. Importantly, the implantation of foreign material may trigger an immune response that can influence the functionality of local cells and inhibit bone repair. Additionally, metal ions released as degradation products can be cytotoxic and trigger a local or systemic immune response [128]. Moreover, many metals are not inherently biocompatible and thereby require surface coating or chemical modification to enhance their surface biocompatibility and reduce host immune reaction [128,129]. Furthermore, as a majority of these scaffolds are nondegradable, a second surgery may be required to remove the implant [129]. These limitations have led to increased interest in magnesium-based implants, which are biodegradable with associated breakdown-products exhibiting the ability to promote bone formation, thereby avoiding the need for implant removal while simultaneously promoting osteogenesis [121]. However, even with degradable scaffolds, manufacturing remains complex and expensive, limiting their production and clinical translation [129].

### 4.2. Bioceramics

Bioceramics are inorganic, non-metallic materials with excellent biocompatibility that can support tissue repair and regeneration. Composed mainly of hydroxyapatite (HA), tricalcium phosphate (TCP), and bioactive glass (BAG), these materials exhibit high osteoconductivity, mimicking the mechanotransductive properties of the native extracellular matrix and resisting corrosion [130]. Their biomechanical properties, specifically porosity, pore interconnectivity, stiffness, and surface features, also directly influence cellular attachment, nutrient diffusion, immune cell recruitment, and the osteogenic effect [131]. These features make them highly versatile and therapeutically applicable to complex fractures and nonunion [132].

In the context of fracture healing, bioceramics modulate the immune pathway through ion release and surface topography. Zhang et al. investigated the influence of calcium release in vitro using calcium phosphate scaffolds with controlled ion-release kinetics and found that sustained Ca^2+^ release activated the calcium-sensing receptor (CaSR) on macrophages, triggering M2 anti-inflammatory polarization [99]. This shift was accompanied by increased IL-10 expression and reduced TNF-α and IL-1β levels. Furthermore, these M2 macrophages also activated downstream osteogenic pathways, specifically BMP-mediated Smad phosphorylation, which enhanced MSC differentiation and bone formation [99]. Chen et al. assessed the utility of calcium-based scaffolds and compared HA, β-TCP, and biphasic calcium phosphate (BCP) scaffolds in a murine intramuscular implantation model [133]. The authors observed that BCP scaffolds promoted macrophage polarization, reduced inducible nitric oxide synthase (a key marker of inflammation), and formed a pro-osteogenic cytokine milieu. This resulted in robust bone formation at 90 days, with 6/7 BCP implants forming mature ectopic bone. Conversely, β-TCP induced a persistent M1-dominant response and failed to initiate osteogenesis [133]. Guo et al. expanded on bioceramic use and its immunomodulatory effects, specifically investigating material surface topography. In a murine subcutaneous implantation model, TCP ceramics with submicron surface features (<1 μm) rapidly induced M2 macrophage polarization, followed sequentially by osteoclast resorption at 2 weeks, and new bone deposition at 4 weeks [100]. This study established a clear timeline highlighting the potential of submicron-textured ceramics to initiate bone healing by impacting the immune cascade [100]. Zhu et al. further evaluated micro/nano-hybrid structured BCP in 12 mm segmental femoral defects in a canine model [134]. These BCP scaffolds downregulated inflammatory pathways, including suppression of TNF signaling and chemokines such as IL-1α, IL-6, and MMP-3 in MSCs [134]. This immunoregulatory profile corresponded with greater bone formation, improved mechanical strength, and a similar modulus as native bone on nanoindentation [134]. Radiographic and histologic assessment confirmed successful defect bridging, and biomechanical testing demonstrated functional healing, with scaffold-treated defects bearing nearly 50% higher compressive loads than controls [134].

Silicate bioceramics have, similarly, exhibited the ability to regulate immune system activity during osteogenesis, and these effects have been demonstrated in both in vivo and in vitro models. In a rabbit femoral condyle defect model, akermanite (Ca_2_MgSi_2_O_7_) bioceramic scaffolds promoted significantly greater bone regeneration than β-TCP control scaffolds at both 8 and 16 weeks, demonstrating successful defect healing with significantly higher new bone volume, coordinated scaffold resorption, and bone ingrowth [102,135]. In vivo murine subcutaneous implantation models also revealed that when compared to β-TCP implants, akermanite scaffolds elicited a significantly reduced inflammatory response with decreased macrophage infiltration, foreign-body giant cell formation, and pro-inflammatory cytokine secretion. This occurred due to suppression of MAPK and NF-κB signaling and induction of macrophage apoptosis through caspase-dependent pathways [135]. The same authors supplemented these findings with evidence that akermanite significantly enhances bone marrow-derived stem cell (BMSC) proliferation and osteoblastic differentiation relative to β-TCP controls, with upregulated alkaline phosphatase (ALP), osteopontin (OPN), and osteocalcin (OCN) expression [102,135].

Beyond calcium and silicate-based ceramics, studies have investigated how alternative bioceramic compositions direct osteoimmune interactions. The influence of ion-specific immunomodulation on bone healing was investigated by Zhang et al. who developed a strontium-substituted submicrometer bioactive glass (Sr-SBG) [136]. In their rat femoral condyle defect model, Sr-SBG was found to increase new bone deposition, suppress IL-6 production, promote M2 polarization, and enhance MSC osteogenic gene expression compared to SBG alone [136]. Li et al. further investigated bioceramic application by using a strontium/copper-doped borosilicate glass (Sr/Cu-BSG) bone cement in both rat and rabbit femoral condyle defect models. The material produced a rapid M1-to-M2 macrophage transition within 3 days, with marked downregulation of IL-1β/IL-6 and upregulation of IL-1 receptor antigen (IL-1Ra) and TGF-β1 [101]. This immune shift was accompanied by a strong angiogenic activation and late osteogenic signaling (i.e., runt-related transcription factor 2 [RUNX-2] and OCN) [101]. In vivo, the Sr/Cu-BSG cement produced a high bone volume fraction, with micro-computed tomography (CT) and histology demonstrating progressive cement degradation and replacement by newly mineralized bone, leading to increased bone volume fraction, bone mineral density (BMD), and bone–implant contact by 16 weeks compared to negative controls [101].

Bioceramics have also been successfully applied to larger animal models. Golafshan et al. treated an equine critical-sized defect with a 3D-printed magnesium phosphate scaffold doped with strontium (MgPSr-PCL30). They reported that the release of Mg^2+^ and Sr^2+^ ions, which are known modulators of macrophage polarization and vascular signaling, was associated with dense vascular infiltration throughout the scaffold’s network and promotion of M2 macrophage polarization [103]. The authors also observed biologically integrated scaffold degradation, with resorption coinciding with infiltration of osseous tissue at 6 months. This was associated with successful healing, evidenced by micro-CT showing enhanced intra-scaffold bone formation compared with empty controls, and bone volume fraction and BMD values approaching those of native bone [103].

While bioceramics present an innovative approach to fracture healing, the therapy is not without limitations. Bioceramics are inherently brittle and possess poor tensile strength and low elasticity, making them unsuitable for load-bearing applications without reinforcement by other materials [84]. Additionally, while bioceramics offer a great platform for ion release, excessive delivery could result in cytotoxicity or oxidative stress [130]. Additionally, when incorporating anti-inflammatory agents or other therapeutics, as well as using calcium or silicate-based ceramics, it is challenging to control the release kinetics over an appropriate time period. Monitoring and reproducing in vivo release behavior across different implantation sites or physiological environments remains a barrier to the widespread integration of such systems [130].

### 4.3. Hydrogels

Hydrogels have the ability to take on the shape of the surrounding structure and encapsulate an area, providing both physical and biochemical support. In contrast, other scaffolds, such as bioceramics and metal implants, are rigid and difficult to conform to irregularly shaped areas. Moreover, hydrogels can be engineered to respond to environmental stimuli, allowing them to degrade and release bioactive therapies in a coordinated manner, whereas other scaffolds may exhibit inconsistent degradation and release rates.

In fracture healing, hydrogels have been shown to support osteogenesis, angiogenesis and immunomodulation. One issue inherent to scaffolds is the risk of immune reaction and inflammation caused by the implantation of a foreign material. To address this limitation, deoxyribonucleic acid (DNA)-based hydrogels have been developed. As DNA exhibits biomimicry and degrades into biocompatible byproducts, these hydrogels minimize immune system activation while engaging in immunomodulation to promote osteogenesis [137]. These hydrogels are formed through cross-linking of deoxyribonucleotide chains and have been shown to upregulate expression of BMP-2 and vascular endothelial growth factor A (VEGFA), key regulators of osteogenesis and angiogenesis [137,138]. Additionally, these hydrogels are able to promote M2 macrophage polarization as well as regulatory T cell differentiation and regulation, leading to an anti-inflammatory microenvironment conducive for bone repair [137].

In addition to immunomodulation, hydrogels also serve as a delivery system for stem cells, cytokines, and osteogenic proteins, such as BMP-2. Their 3D structure can promote the proliferation and differentiation of MSCs, providing a matrix for osteogenesis. Hydrogels, combined with growth factors or cytokines, have been previously successful in healing alveolar defects, critical-sized defects, and nonunions, highlighting their value in complex bone repair scenarios [104,105,139,140]. For example, a DNA hydrogel containing Apt02-tFNA, a DNA oligonucleotide with similar activity to VEGFA, was able to enhance bone healing in a rat critical-sized calvaria defect model [104]. There was increased expression of OPN, OCN, and RUNX2, as well as increased BMD and new bone volume at the defect site [104]. Furthermore, one significant risk factor for fracture nonunion is infection and the corresponding inflammatory environment. Yu et al. found that microRNA-708-5p, which suppresses the Wnt/β-catenin signaling pathway, is upregulated in infected nonunions [105]. To target this inflammatory pathway, a hyaluronic acid hydrogel loaded with antagomiR-708-5p was developed, demonstrating sustained release of antagomiR-708-5p as well as antibacterial activity against Staphylococcus aureus, thereby reducing biofilm formation [105]. In an in vivo murine model with a transverse femoral shaft fracture infected with Staphylococcus aureus, this hydrogel resulted in improved bone volume and increased expression of osteogenic proteins compared to controls [105]. The bone regenerative capacity of hydrogels can be further enhanced by integrating stem cells. Ingalve et al. implanted a hydrogel cellularized with BMSCs into a sheep iliac crest defect model, demonstrating that the integration of stem cells within a hydrogel can increase bone volume fraction and histologically improve bone formation compared to acellular hydrogels [141].

Despite their potential, hydrogel use is limited by their mechanical weakness, inconsistent degradability, and cytotoxicity. Hydrogels often exhibit low stiffness and toughness, making them unsuitable for weight-bearing regions where mechanical support is needed, and often lose their mechanical strength after implantation [142]. While the use of synthetic polymers and cross-linking agents can enhance mechanical strength, their use may lead to cytotoxic byproducts, negatively affecting biocompatibility [142,143]. Additionally, in areas with high mechanical stress, hydrogels may degrade more rapidly than anticipated, causing treatment failure before sufficient repair is achieved [144].

### 4.4. Nanoparticles and Microspheres

Nanoparticles (NPs) and microspheres have also emerged as an innovative delivery system capable of enhancing skeletal tissue repair through targeted modulation of immune responses and the release of bioactive molecules [145,146,147]. Typically ranging from 1 to 100 nm, NPs provide a high surface-area-to-volume ratio that allows for efficient encapsulation of cytokines, growth factors, or small-molecule drugs [148]. Commonly employed materials include bioactive ceramics (HA, calcium phosphate), polymers (PLGA, chitosan), liposomes, and metallic oxides (silica, titanium dioxide, zinc oxide) [149]. Microspheres (1–100 µm) have similarly been employed for direct modulation of the immune microenvironment [106,150]. Overall, microspheres have proven better for localized and sustained release within a confined space, whereby their larger size prevents systemic diffusion and allows for delivery over days to weeks [147]. In contrast, NPs enable better cellular uptake and tissue penetration, allowing for targeted intracellular delivery to immune or progenitor cells such as macrophages or MSCs [147,150]. Therefore, researchers employ NPs at a cellular and molecular level, while using microspheres in regional modulation of the inflammatory microenvironment [147,150].

In the context of fracture healing, Xiao et al. developed a fracture-targeted NP drug delivery system to modulate the immune microenvironment [106]. By loading AR28, a small-molecule inhibitor of glycogen synthase kinase-3 beta (GSK3β), the authors observed decreased TNF-α and IL-1β signaling and a transition towards an M2 phenotype. This promoted greater callus formation, bone volume fraction, and mechanical strength compared to empty controls [106]. Alternatively, Song et al. developed a poly(lactic-co-glycolic)/manganese dioxide (PLGA/MnO_2_) microsphere system for bone-defect repair loaded with BMP-2 [108]. The MnO_2_ neutralized the acidic, reactive oxygen species (ROS)-rich injury microenvironment and generated O_2_/Mn^2+^ ions, thus reducing the number of pro-inflammatory cells at the injury site. This immune-reprogramming enhanced BMP-2-driven osteogenesis and improved bone repair outcomes [108]. With the evolution of bioengineering approaches, biomimetic anti-inflammatory nanoparticles (BANC) have emerged as an innovative approach to promote fracture healing. Yin et al. fabricated BANCs by coating NPs with lipopolysaccharide-treated macrophage cell membranes containing pro-inflammatory cytokine receptors, thereby creating a locally deliverable competitive cytokine inhibitor [107]. In their mouse femoral defect model, this BANC rapidly neutralized excess TNF-α and IL-6 in the early inflammatory phase and induced robust M2 macrophage polarization. This resulted in healed defects with significantly enhanced osteogenesis at four weeks, with greater new bone formation, collagen deposition, and mineralized tissue compared to controls [107].

Despite their promise, the use of NPs and microspheres have limitations to their clinical use. Challenges include cytotoxicity, unpredictable biodistribution, and unwanted immune responses [148]. There have been attempts to address these issues through surface modification and functionalization with biocompatible molecules. For instance, modifying the surface composition, superficial charge, size, and shape of the particle can substantially alter uptake efficiency and overall safety [148]. Small particles (<10 nm) are more likely to traverse cellular membranes and have been associated with oxidative stress, DNA damage, and cellular apoptosis, largely due to cationic surface interactions with negatively charged cellular membranes and plasma proteins [151]. To improve targeting and reduce off-target effects, surface peptides can act as specific targeting ligands, resulting in safe and targeted component release [151]. Additionally, to combat particle recognition, “stealth” coating of small particles using platelets or red blood cell-derived polysaccharides or biomimetic membranes has been found to reduce immune recognition, limit cellular uptake, and improve bioactivity [148]. Human serum albumin has also become increasingly popular due to its abundance, biocompatibility, and ability to heighten tissue targeting while reducing toxicity [152]. In summary, while emerging strategies continue to mitigate these limitations, further work is needed to optimize safety and enable broader clinical use of microspheres and NPs.

## 5. Integration of Immunomodulation with Molecular and Cellular Therapies

In addition to their immunomodulatory properties, scaffolds can be utilized to enhance bone regeneration by delivering osteogenic cells and gene vectors. As mentioned previously, scaffolds provide an osteoconductive surface for bone regeneration but lack sufficient osteoinductive signals, particularly in nonunion. As scaffolds are capable delivery systems, optimization of their immunoregulatory properties may further enhance healing, particularly in recalcitrant nonunions with pathologic immune microenvironments.

### 5.1. Stem Cell Therapy

Stem cells are a promising therapy being explored to promote bone healing. MSCs are multipotent cells that can self-renew and are found in various tissues, including adipose tissue, placental tissue and bone marrow [153]. MSCs have the potential to differentiate into osteocytes or chondrocytes, making them key therapeutic agents for skeletal tissue repair. The addition of exogenous stem cells to scaffolds can increase the number of functional stem cells at the site of injury, improving tissue regeneration [154]. Beyond their regenerative potential, MSCs also have immunomodulatory effects that can assist in tissue repair [155]. MSCs interact with various immune cells through direct cell-to-cell contact and paracrine signaling. Through the upregulation of ICAM-1 and VCAM-1 cell adhesion molecules, MSCs have been shown to suppress T-lymphocyte activation, and directly interact with NK cells to modulate their cytotoxic activity [156,157]. Further, prostaglandin E2 secreted by MSCs promotes macrophage polarization towards the anti-inflammatory M2 phenotype [158]. While MSCs and functionalized scaffolds are commonly discussed as independent therapeutics, increased research has shown that MSC survival and function are significantly impacted by the immunologic properties of the scaffold microenvironment [159]. Immunomodulatory scaffolds are able to locally suppress excessive inflammatory signaling and enrich regulatory immune cells. This allows for implantation of MSCs into a regulated environment, preventing rapid cell loss and impaired osteogenic differentiation, which commonly results when cells are implanted into a pro-inflammatory environment [159,160]. Pro-inflammatory cytokines such as TNF-α, IL-1β, and IFN-γ can induce MSC apoptosis, promote senescence, or cause a phenotypic change toward a fibroblastic state, ultimately limiting MSCs’ regenerative capacity [161,162,163]. Conversely, anti-inflammatory microenvironments have been shown to result in MSC secretion of prostaglandin E2, TGF-β, and IL-10, sustaining macrophage M2 polarization and suppressing cytotoxic T-cell activity [159,160]. Similarly, scaffolds that buffer oxidative stress, normalize pH, or release immunoregulatory ions, such as magnesium, calcium or strontium, reduce cellular stress responses that impair MSC engraftment and differentiation [159]. Ultimately, immunomodulatory scaffolds can modulate the therapeutic environment and provide a synergistic effect in bone repair.

When the native local biology and traditional therapies, such as metal implants and bone grafts, are inadequate, stem cells can be integrated to enhance the bone’s healing capacity [164]. MSCs are recruited to the fracture site through pro-inflammatory cytokines, including TNF-α, IL-1α, and IL-1β, secreted by local immune cells [165]. Upon migration to the fracture site, MSCs differentiate into osteoblasts and exert immunomodulatory effects on surrounding cells. Through the integrated action of INF-*γ* and expression of inducible nitric oxide synthase, MSCs inhibit pro-inflammatory T-cell activity and oppose the inflammatory environments [166]. Furthermore, MSCs promote M2 polarization and inhibit chemotactic signals through prostaglandin-dependent pathways, thereby further promoting the resolution of the local inflammatory state [166]. For fracture nonunion, MSC therapy has been shown to accelerate and enhance short-term postoperative healing clinically. In a meta-analysis of 21 studies involving nearly 900 patients, it was found that MSCs shortened the time to nonunion healing and increased healing rates at 3 and 6 months compared to controls [109]. Furthermore, in a study of 10 nonunion patients, Ismail et al. noted earlier radiographic signs of healing and improved functional outcomes in the early postoperative period in patients treated with BMSCs [110]. In pre-clinical models, MSCs have also been used in combination with scaffolds to improve fracture healing and enhance immunomodulation of the fracture environment. Using a rat femoral defect model, Li et al. implanted silicate nanoplatelets with BMSCs and observed improved bone formation histologically when compared to defects treated with nanoparticles alone [111]. Additionally, immunohistochemical staining showed increased M2 macrophages around the defect site and decreased M1 macrophages compared to nanoplatelets alone, indicating that the immunoregulatory properties of MSCs likely played a role in fracture repair [111].

Despite holding significant promise, wide-scale applicability of stem cell therapy is hindered by various challenges. Stem cell harvesting can lead to iatrogenic pain and often requires a secondary procedure. To mitigate this, methods have been developed to isolate MSCs from lipoaspirate or via allogeneic stem cells [165,167]. Although allogeneic MSCs are appealing to avoid a secondary procedure in patients, their use introduces the potential for alloreactivity. Moreover, while MSCs are screened for infectious agents, they have been shown to contain latent viral DNA, such as Parvovirus B19, which could theoretically infect patients implanted with these cells [168,169]. Furthermore, MSCs also exhibit potential tumorigenicity, with evidence suggesting that they are involved in tumor growth and metastasis [170,171]. Using mouse models, Corcoran et al. demonstrated that BMSCs can help facilitate the spread of breast cancer into bone marrow, while Suzuki et al. showed that thymus-derived MSCs can increase the volume of tumors when co-implanted with B16-LacZ cancer cells. It must be noted that the potential tumorigenicity of MSCs has been predominantly shown to occur in the setting of co-implantation with cancer cells rather than their direct malignant transformation [170,171,172]. As regenerative medicine continues to advance, stem cell-based therapies are being integrated with other therapeutic strategies such as scaffolds or gene therapy; however, concerns regarding safety and tumorigenicity must be addressed to facilitate their clinical translation.

### 5.2. Gene Therapy

Genetic modification of MSCs or direct viral delivery to fracture sites enables sustained, localized bioactivity without the need for repeated protein administration. Viral vector-mediated gene delivery using adenoviral (AD), lentiviral (LV), and adeno-associated viral (AAV) vectors remains the predominant method of achieving stable gene expression [173].

Gene therapy has been integrated with scaffolds and stem cells to enhance fracture healing. For example, Vakhshori et al. demonstrated the successful healing of critical-sized femoral defects using a TCP/HA scaffold to deliver human adipose-derived stem cells transduced with an LV vector to express BMP-2 directly at the defect site [112]. Additionally, gene therapy alone and in combination with various scaffolds have demonstrated immunomodulatory effects, leading to improved bone regeneration. In using a PLGA scaffold seeded with MSCs transduced with BMP-2 and VEGF, Lin et al. demonstrated successful healing in a rabbit femoral critical-sized defect [112]. The scaffold containing genetically modified cells facilitated bony bridging at 2 weeks and complete radiographic healing at 8 weeks post implantation in 13/13 defects. Furthermore, this therapy improved bone formation in a dose-dependent manner, with higher doses resulting in more complete bridging as assessed via micro-CT [113]. Moreover, using gene therapy to target the immune cascade in fracture healing is another emerging strategy. For example, AAV-mediated IL-4 delivery was found to polarize macrophages toward the M2 phenotype and, as confirmed by micro-CT and histological analysis, significantly reduce bone resorption [66]. The IL-1 receptor pathway has also been investigated as a potential therapeutic target to improve fracture healing, as IL-1β inhibits osteogenesis and bone mineralization through downregulating the expression of ALP and integrin-binding sialoprotein (IBSP). By leveraging this pathway, Lackington et al. observed that NPs containing IL-1Ra pDNA loaded onto a collagen-HA scaffold were able to restore the expression of ALP and IBSP within BMSCs in vitro, thus promoting osteogenesis and calcium deposition [174]. Panos et al. expanded upon these findings by using combination therapy to heal a rodent femoral critical-sized defect, demonstrating that direct IL-1Ra gene delivery to the defect site achieved bony bridging with a 90% lower dose of rhBMP-2. Given that the physiologic doses of rhBMP needed to trigger bone healing have been shown to result in heterotopic ossification, an increased risk of seroma formation, and carcinogenic potential, this study’s finding that modulation of the local immune microenvironment enables bone regeneration using lower doses of growth factors may have clinical potential [175,176]. Despite some early success, studies in clinically relevant animal models are needed [114].

## 6. Future Directions

The future of osteoimmunology-based therapy in the context of bone repair is highly promising. Researchers continue to develop strategies to actively program the local immune environment with spatiotemporal control [177,178]. For example, scaffolds and bioceramics capable of staged release of Ca^2+^ and Si^4+^ ions continue to be developed to guide the M1-to-M2 macrophage transition, co-deliver IL-10 and TGF-β mimetics, and leverage surface topographies to induce coordinated macrophage and osteoclast–osteoblast coupling during fracture healing [177,178]. Additionally, “smart” responsive systems, for example, ROS-, pH-, or enzyme-responsive hydrogels and coatings, represent an evolving area of investigation. These therapies are designed to release anti-inflammatory drugs and biomolecules selectively at sites where inflammation is high [179,180]. ROS-cleavable and bacteria-sensing systems have already shown progress in this area [181]. Another rapidly advancing area involves apoptotic and exosomal vesicles derived from MSCs or engineered macrophages. These vesicles, when packaged within ceramics or hydrogels, can deliver miRNAs that drive macrophage M2 polarization and enhance osteogenic differentiation, offering an innovative, cell-free immunotherapy platform [182,183,184].

There are also significant translational barriers between pre-clinical models and clinical application. Notably, osteoimmunology research as applied to fracture healing remains limited, with current research primarily focusing on in vitro and small animal models. Naturally, immune phenotypes are often highly variable, differing by species, age, and comorbidities [185,186,187]. As such, the effect size of positive outcomes observed in small animals may over-estimate efficacy in large animal models or humans [188]. Specifically, there are significant interspecies differences in immune system composition, hindering the progression to human-based studies. Relative to humans, mice demonstrate reduced relative levels of circulating neutrophils, increased lymphocyte proportions, and macrophages governed by distinct molecular activation cues, all of which play central roles in fracture repair [189]. As a result, osteoimmunologic studies that discuss the immune modulation mechanisms of scaffolds may not accurately depict the underlying modulatory effects in humans, and may be targeting mechanisms that are not present or are functionally different in humans. In response, humanized immune system models in rats and mice have been developed to better reflect human conditions, but these are rarely used in the context of osteoimmunology and fracture union [190,191]. Further, many nonunion models create segmental defects in animals, as these are the most reproducible. Conversely, modeling an atrophic nonunion model is challenging as it lacks biological activity that is difficult to replicate in a research setting. Despite these barriers, researchers have aimed to induce atrophic nonunion through infected nonunion models or through genetic modifications to deplete osteogenic cell populations necessary for fracture repair [105,192]. To address many of these limitations, more advanced large animal models are currently being developed to better represent human conditions. For example, large animal models such as canines have been shown to better model the mechanical strength of bone in humans compared to rat models, while non-human primate models have been shown to more accurately reflect the biomechanical environment of fracture healing in humans compared to canines and pigs [193]. Additionally, these large animal models demonstrate osteon remodeling by Haversian systems which is absent in rats and mice, allowing them to more closely resemble human bone remodeling [193]. We must also note that humans and small animal models have different fracture healing timelines which may further diminish clinical translatability. Rabbits and rats exhibit rapid bone healing and shortened metabolic cycles relative to humans, whereas larger animal models display medullary-based healing over a prolonged timeframe, more closely mirroring human fracture repair [193]. Therefore, continued research is needed to develop accurate large animal models that better represent human fracture healing and the osteoimmune environment associated with fracture repair. Finally, there are also safety concerns associated with immune manipulation. Use of viral vectors requires continued monitoring of biodistribution, persistence, and immunogenicity, while hydrogel-based systems must be evaluated for potentially cytotoxic degradation byproducts [194,195].

While challenges remain, particularly in variability of immune phenotypes, species-dependent responses, and safety considerations with gene delivery and degradable polymers, the trajectory of osteoimmunology-based therapy in bone repair shows great potential [185,186,187]. With new and emerging advances in single-cell immune profiling, patient-specific biomaterial design, and more rigorous translational safety frameworks, osteoimmunomodulatory therapies continue to move closer to clinical application.

## 7. Concluding Remarks

Osteoimmunology is a rapidly growing field and the understanding of the complex interplay between the skeletal and immune systems is crucial for the development of novel solutions to conditions such as fracture nonunion, a condition characterized by chronic inflammation and a pro-inflammatory environment. Bone regeneration is profoundly influenced by the immune system, and effective healing requires modulation of the immune system, commonly through promotion of anti-inflammatory pathways as well as inhibition of excessive inflammation. The integration of multiple therapeutic agents, such as scaffolds seeded with genetically modified stem cells, offers a multimodal approach to attain immunomodulation and osteogenesis. However, continued research is needed to optimize material biocompatibility, mitigate adverse host immune reaction, and promote tissue integration to ensure continued skeletal tissue regeneration.

## Figures and Tables

**Table 1:** Summary of recent therapeutic studies for fracture healing.

Study	Population/Model	Intervention	N	Outcomes	Reference
Fracture Studies					
Liu et al., 2021	Rat critical-sized calvaria defect model	Polymer scaffold (3D-printed PCL scaffold integrated with electrospun microfibers)	40	-3D-printed PCL scaffolds with electrospun microfibers promoted macrophage polarization toward the M2 phenotype compared to nanofiber or PCL-only controls. - M2 polarization occurred via activation of the PI3K/AKT signaling pathway. - Defects were healed with increased angiogenesis, higher BV/TV, BMD, and accelerated bone regeneration on micro-CT and histologic analysis.	[91]
Long et al., 2023	Rat femoral defect model	Polymer scaffold (3D-printed PLGA scaffold incorporating black phosphorus [PLGA/BP])	12	-PLGA/BP scaffolds recruited macrophages and promoted M2 polarization (↑ IL-10, ↓ TNF-α, IL-6). -Fractures were healed with micro-CT, showing significantly increased bone volume, BV/TV, trabecular thickness, and bone mineral density.	[83]
Taraballi et al., 2016	Rat subcutaneous implantation model	Polymer scaffold (collagen with chondroitin sulfate [CSCL])	6	-In vitro studies showed that CSCL scaffolds resulted in inhibition of LPS/CD44/NF-kB, leading to a decrease in pro-inflammatory molecules (TGF-β, Arg, MRC1, and IL-10) and an increase in anti-inflammatory markers (TNF-a and iNOS). -In vivo implantation of the CSCL scaffold resulted in decreased expression of CD44 and pro-inflammatory markers (TNF-α, iNOS, IL-12β, IL-1β, and MMP-1).	[92]
Chen et al., 2025	Rat femoral defect model	Metal scaffold (MgZnYNd magnesium alloy rod)	54	-A MgZnYNd magnesium alloy rod was implanted into a rat femoral defect model. -It demonstrated improved bone volume when compared to titanium as well as increased periosteal progenitor cell-derived osteogenesis, periostin and COL1A1 secretion via RNA sequencing, and enhanced M2 polarization via increased IL-10 expression.	[93]
Pitchai et al., 2022	In vitro macrophage studies	Metal scaffold (titanium)	39	-Systematic review covering 39 articles. -Titanium surface modifications that increase hydrophilicity and surface roughness can cause a shift in macrophages toward the M2 phenotype, which has the potential of improve osteogenesis and osseointegration.	[94]
Gehrke et al., 2018	Insertion of titanium screws into rabbit tibia	Metal scaffold (sandblasted and acid-etched [SLA] titanium implants with a calcium–magnesium coating)	10	- SLA titanium implants were coated with CaMg and inserted into the tibias of rabbits. - These CaMg-coated implants displayed a higher degree of bone organization with a higher bone-to-implant contact percent and increased new bone formation compared to SLA implants alone, demonstrating the utility of CaMg in the osseointegration of implants.	[95]
Liu et al., 2024	Rat femoral defect model	Metal scaffold (metal–phenolic network on titanium implant)	36	- A metal–phenolic network was utilized as a multifunctional nanocoating on titanium implants. - There was increased M2 polarization as well as improved bone volume and new bone formation about the bone–implant interface compared to titanium alone.	[96]
Pobloth et al., 2018	Ovine mid-diaphyseal tibial defect model	Metal scaffold (3D titanium mesh)	27	- 3D titanium mesh scaffolds with various levels of stiffness were implanted within a sheep tibia defect model. - Less stiff implants resulted in lower stress shielding. - Within subjects treated with implants of lower stiffness, there was earlier bony bridging radiographically, with 3/6 of the subjects showing complete bony bridging at 8 weeks compared to 0/6 in the subjects treated with stiffer implants. -Less stiff implants also demonstrated greater bone formation on histology.	[97]
Han et al., 2020	Rat femoral condyle defect model	Metal scaffold (magnesium alloy implant)	16	- Magnesium alloy implant was able to release Mg, Ca, and Zn ions. -Magnesium alloy implant resulted in increased type II collagen deposition at the bone–implant interface compared to titanium implants. - Two weeks post implantation, subjects treated with the magnesium alloy implant and those treated with a titanium implant experienced a similar relative number of osterix-positive cells, but by 4 weeks, the magnesium alloy implant resulted in nearly double the relative quantity compared to titanium implants.	[98]
Zhang et al., 2021	Ectopic implantation of Biphasic calcium phosphate (BCP) ceramics in mice	Bioceramic (calcium phosphate)	12	- Controlled Ca^2+^ release from calcium phosphate bioceramics activates CaSR on macrophages. - CaSR activation promoted M2 macrophage polarization, increasing IL-10 while decreasing TNF-α and IL-1β expression. - Conditioned medium from these M2 macrophages significantly enhanced osteogenic differentiation of MSCs, upregulating Smad/BMP pathways. - In vivo, Ca^2+^-releasing ceramics generated a more robust osteogenic microenvironment, with increased early vascularization and higher mineralized bone volume compared to bioceramics containing a CaSR blocking agent.	[99]
Guo et al., 2021	Ectopic implantation of tricalcium phosphate (TCP) ceramics in FVB mice	Bioceramic (TCP)	40	- Submicron-structured TCPs rapidly recruited macrophages. - TCPs induced early M2 polarization within 7 days, unlike micron-scale ceramics. - Early depletion of macrophages on day 1 blocked M2 polarization, osteoclast formation, and bone formation; osteoclastogenesis occurred mainly in week 2, followed by new bone deposition starting around week 4. - Bioceramic surface topography orchestrates the immune-to-bone healing cascade.	[100]
S. Li et al., 2023	Rat and rabbit femoral condyle defect models	Bioceramic (strontium/copper-doped borosilicate glass [Sr/Cu-BSG] bone cement)	36 rats, 72 rabbits	- Sr/Cu-borosilicate glass bone cement shifted macrophages toward the M2 phenotype, enhanced angiogenesis (↑VEGF/FGF), and produced the greatest bone regeneration. - This scaffold demonstrated the highest bone volume fraction, BMD, bone–implant contact, and mineral apposition rate, resulting in superior immunomodulatory, pro-vascular, and osteogenic capacity compared to all other formulations and CaSO_4_ control.	[101]
Huang et al., 2018	Rabbit femoral defect model	Bioceramic (calcium magnesium silicate bioceramic)	32	- A calcium magnesium silicate bioceramic was compared to β-TCP and was found to enhance bone formation, improve stem cell differentiation and upregulate osteogenic protein expression. - In vitro, when co-cultured with BMSCs, the calcium magnesium silicate bioceramic upregulates ALP, OPN, OCN, and IBSP expression compared to β-TCP. - In vivo, the calcium magnesium silicate bioceramic demonstrated increased bone mineralization and bone volume with faster degradation compared to β-TCP.	[102]
Golafshan et al., 2020	Equine critical-sized tuber coxae defect mode	Bioceramic (magnesium phosphate doped with strontium in a polycaprolactone matrix [MgPSr-PCL30])	8	- The scaffold released Mg^2+^ and Sr^2+^ ions, promoting M2 macrophage polarization and angiogenesis. - The scaffold underwent biologically integrated degradation (~15% resorption) while becoming infiltrated by endothelial cells, osteoblasts, and osteocytes. - On micro-CT, there was greater new bone formation in subjects treated with the experimental scaffold compared to empty controls. - BMD and bone volume fraction in the experimental group approached native bone, demonstrating effective bridging of a weight-bearing critical-sized defect.	[103]
Han et al., 2024	Rat cranial defect model	Hydrogel (DNA)	Not specified	- A hydrogel containing polymer-modified DNA hydrogel and aptamer02 (an oligonucleotide that induces angiogenesis) was developed. - In vitro, BMSCs were cultured with the hydrogel, showing downregulation of genes within the IL-17 and TNF pathways, demonstrating the hydrogel’s anti-inflammatory effects. - When implanted into a rodent critical-sized defect, researchers observed increased bone formation, new bone volume, and bone mineral density within the defect site compared to control PCL scaffolds.	[104]
Yu et al., 2025	Rat femoral nonunion model	Hydrogel (hyaluronic acid)	90	- MicroRNA-708-5p was found to be upregulated in infected nonunions. - The multifunctional hyaluronic acid hydrogel loaded with antagomiR-708-5p showed antibacterial activity against S. aureus, reducing biofilm formation. - There was sustained release of antagomiR-708-5p in vitro. - In a femur nonunion murine model, the hydrogel loaded with antagomiR-708-5p showed improved bone volume and increased expression of osteogenic proteins compared to the hydrogel alone.	[105]
Xiao et al., 2024	Mouse femoral fracture model	Nanoparticle (AR28)	24	-Bone-targeting nanoparticles were loaded with AR28, a small-molecule GSK3β inhibitor known to enhance osteogenesis and modulate macrophage inflammatory signaling. - The macrophage uptake of AR28 promoted M2 polarization, reduced pro-inflammatory cytokine secretion (IL-1β, TNF-α), and increased IL-10 expression. - There was improved callus formation and bone volume fraction, increased mechanical strength of healing bone, and improved cartilage-to-bone progression consistent with enhanced endochondral ossification compared to nanoparticles alone.	[106]
Yin et al., 2020	Mouse femoral defect model	Biomimetic anti-inflammatory nano-capsule (BANC)	20	-BANC neutralized excess TNF-α and IL-6 through cytokine receptor-decorated macrophage membranes. - Early inflammatory cell activity was inhibited and robust M2 macrophage polarization was induced. - There was significantly improved new bone formation at 4 weeks, with 2–3 times greater type I collagen and mineralized tissue compared to controls.	[107]
Q. Song et al., 2024	Rat critical-sized calvaria defect model	Microsphere (H–MnO)	35	- Microspheres respond to the inflammatory microenvironment. - H–MnO microspheres released anti-inflammatory and osteogenic cues, decreased M1 markers, increased M2 markers, enhanced MSC osteogenesis, and significantly improved new bone formation and bone mineral density in critical-sized defects compared to microsphere without H–MnO.	[108]
Cui et al., 2025	Patients with nonunion	Stem cell	866	- Meta-analysis features 866 nonunion patients who were treated with MSC therapy. - There were, overall, significantly faster healing rates at 3 and 6 months in patients treated with MSCs, but no difference was seen compared to traditional methods past 6 months. - Patients who were treated with MSCs + scaffold saw improved healing rates past 12 months after treatment. - Treatment with MSCs was found to reduce time to union with MSCs + scaffold observed to have an enhanced effect on union time.	[109]
Ismail et al., 2016	Patients with atrophic nonunion	Stem cell	10	- In the early postoperative period, patients treated with MSCs showed greater functional improvement and faster bone growth on radiographs, suggesting that patients treated with MSCs reached union clinically at a faster rate.	[110]
T. Li et al., 2018	Rat femoral defect model	Stem cell	Not specified	- BMSCs were implanted into a murine critical-sized bone defect with silicate nanoplatelets. - In vitro, expression of osteogenic genes, including ALP, RUNX2, OCN, OPN, IBSP, and COL-1 in MSCs, was upregulated in a dose-dependent fashion with the nanoplatelets. - On histology, MSCs + nanoplatelets demonstrated apparent bone formation at the implantation site, whereas nanoplatelets alone showed no bone formation. -Immunohistochemical analysis revealed increased M2 macrophage polarization and decreased M1 macrophages at the defect site compared to nanoplatelets alone.	[111]
Vakhshori et al., 2020	Rat critical-sized femoral defect model	Gene therapy	38	- A phosphate/HA scaffold was seeded with adipose-derived stem cells transduced using a lentiviral vector to overexpress BMP-2 and was implanted into a rat critical-sized defect. - The experimental group demonstrated improved radiographic healing, increased histologic bone formation, and increased bone volume compared to negative controls and was comparable to positive controls. - Biomechanical testing demonstrated no significant difference between the experimental group and the positive controls.	[112]
Lin et al., 2010	Rabbit femoral defect model	Gene therapy	48	- BMSCs were transduced using a baculovirus to express BMP-2 or VEGF. - MSCs expressing BMP-2 and MSCs expressing VEGF were loaded onto PLGA scaffolds and implanted into a rabbit femoral defect. -Individuals treated with scaffolds containing genetically modified MSCs demonstrated improved radiographic healing at 4 and 8 weeks post implantation as well as improved bone formation on histology compared to controls. - Treatment with genetically modified MSCs resulted in enhanced bone bridging on microCT and improved biomechanical properties.	[113]
Panos et al., 2023	Rat critical-sized femoral defect model	Gene therapy	344	- IL-1 blockade reduced IL-1β, TNF-α, and early macrophage-driven catabolism. - IL-1Ra gene transfer along with administration of rh-BMP increased callus size, improved bridging, and increased biomechanical strength, and resulted in more uniform mineralization and greater trabecular organization compared to defects treated with rh-BMP alone.	[114]
H. Kim et al., 2025	LPS-induced calvaria osteolysis model	Gene therapy	24	- AAV-IL-4 delivery produced sustained IL-4 expression and increased M2 polarization. - There were reduced inflammatory cytokines, improved callus maturity, enhanced trabecular formation and bone volume, and increased biomechanical strength compared to negative controls.	[115]

## Data Availability

Not applicable.
